# IGF-1 Induces GHRH Neuronal Axon Elongation during Early Postnatal Life in Mice

**DOI:** 10.1371/journal.pone.0170083

**Published:** 2017-01-11

**Authors:** Lyvianne Decourtye, Erik Mire, Maud Clemessy, Victor Heurtier, Tatiana Ledent, Iain C. Robinson, Patrice Mollard, Jacques Epelbaum, Michael J. Meaney, Sonia Garel, Yves Le Bouc, Laurent Kappeler

**Affiliations:** 1 Sorbonne Universités, UPMC Univ Paris 06, INSERM, UMRS 938, Centre de Recherche St-Antoine (CRSA), Paris, France; 2 MRC, National Institute for Medical Research, Division of Molecular Neuroendocrinology, London, United Kingdom; 3 Universités Montpellier 1 and 2, CNRS, UMR 5203, Institut de Génomique Fonctionnelle, Montpellier, France; 4 Sorbonne Paris Cité, Université Paris Descartes, INSERM, UMR 894, Centre de Psychiatrie et Neurosciences, Paris, France; 5 McGill University, Douglas Mental Health University Institute, Montreal, Canada; 6 Ecole Normale Supérieure, Institut de Biologie de l'ENS, IBENS, Paris, France; University of Cordoba, SPAIN

## Abstract

Nutrition during the perinatal period programs body growth. Growth hormone (GH) secretion from the pituitary regulates body growth and is controlled by Growth Hormone Releasing Hormone (GHRH) neurons located in the arcuate nucleus of the hypothalamus. We observed that dietary restriction during the early postnatal period (i.e. lactation) in mice influences postnatal growth by permanently altering the development of the somatotropic axis in the pituitary gland. This alteration may be due to a lack of GHRH signaling during this critical developmental period. Indeed, underfed pups showed decreased insulin-like growth factor I (IGF-I) plasma levels, which are associated with lower innervation of the median eminence by GHRH axons at 10 days of age relative to normally fed pups. IGF-I preferentially stimulated axon elongation of GHRH neurons in *in vitro* arcuate explant cultures from 7 day-old normally fed pups. This IGF-I stimulating effect was selective since other arcuate neurons visualized concomitantly by neurofilament labeling, or AgRP immunochemistry, did not significantly respond to IGF-I stimulation. Moreover, GHRH neurons in explants from age-matched underfed pups lost the capacity to respond to IGF-I stimulation. Molecular analyses indicated that nutritional restriction was associated with impaired activation of AKT. These results highlight a role for IGF-I in axon elongation that appears to be cell selective and participates in the complex cellular mechanisms that link underfeeding during the early postnatal period with programming of the growth trajectory.

## Introduction

Normal development of both cognitive and somatic functions requires suitable nutrition of the developing fetus and newborns during the perinatal period [1]. Indeed, insufficient food supplies during the early postnatal period affect body growth as well as the risk of developing cardiovascular and metabolic diseases in adulthood. This is in agreement with the developmental origin of health and adult diseases (DOHaD), particularly after intrauterine growth retardation, which accounts for 5% of births [2]. This programming of adult diseases, which has been validated in various animal models as well as in human populations [1,3,4,5], further highlights the importance of mechanisms that link the sensing of nutritional state to a shift in the developmental trajectory, which is strongly integrated with that of linear growth.

Body growth is controlled by a subset of neurons located in the hypothalamus that regulates the secretion of Growth Hormone (GH), part of the somatotropic neuro-endocrine axis. The developmental settings of the somatotropic axis are highly sensitive to early postnatal nutrition [6,7], suggesting that this subset of hypothalamic neurons is particularly sensitive to nutritional supply during the perinatal period. Growth programming is also associated with the insulin-like growth factor I (IGF-I) receptor signaling pathway [7]. Indeed, the permanent growth delay phenotype resulting from caloric nutritional restriction during lactation [6] can be genetically mimicked by tissue-specific heterozygous inactivation of the IGF-1 receptor (IGF-1R) in the central nervous system [7].

GH is secreted from pituitary somatotroph cells under the regulation of two hypothalamic neurohormones, Growth Hormone Releasing Hormone (GHRH) that stimulates GH secretion, and Somatostatin (SRIH) that inhibits it. Our previous reports suggest that GHRH neurons located in the arcuate nucleus of the hypothalamus may be a key element that influences programming of the growth trajectory [6,7]. Indeed, we previously observed that the heterozygous invalidation of IGF-1R alters the development of the somatotropic axis, which is associated with a decrease of GHRH immune-reactivity in the median eminence at 10 days of age and a specific permanent pituitary hypoplasia of GH-producing somatotroph cells by 20 days of age. These findings are in agreement with previous studies that highlighted a strong implication of GHRH in the stimulation of proliferation and differentiation of somatotroph cells during the early postnatal period through an induction of Pit-1in developing pituitary [8]. Moreover, our previous report indicates that nutritional restriction during lactation in wild type pups, which decrease circulating levels of IGF-I, similarly induces a permanent pituitary hypoplasia in somatotroph cells by 20 days of age that is preceded by a transient decrease of GHRH expression level at 10 days of age. These data indicate a physiological relevance for IGF-I signalization on the development of the somatotropic axis. Notably, they suggest that the reprogramming of the somatotropic axis by nutritional restriction foremost involves GHRH neurons during the first week of life, when the GHRH neurons of the hypothalamus are still developing, through an underlying mechanism that remains to be deciphered. The current study aims to highlight how IGF-I acts on GHRH neurons during this critical window.

Developing arcuate neurons may be highly sensitive to IGF-I signaling because the mediobasal hypothalamus is strongly enriched for IGF-I receptors [9,10]. IGF-I exerts strong proliferative and anti-apoptotic effects [11]. In developing brain, IGF-I signaling is also strongly associated with cellular morphogenesis: high IGF-I levels in the brain are associated with long and complex neuronal dendritic arborization [12,13,14]. In addition, IGF-I may also determine axon fate through the IGF-1Rgc isoform during the early phase of neuronal differentiation [15,16,17]. During the latter phase of neuronal development, IGF-1R signaling is also implicated in the control of axon guidance in the lateral olfactory bulb [18]. A few reports have studied the role of IGF-I in axon elongation: Ozdinler *et al*. have shown that IGF-I can stimulate axonal outgrowth in neurons [19], whereas other reports suggest that all neurons do not display the same sensitivity to IGF-I [18,20]. Here, we determined how undernutrition during the early postnatal period affects the development of GHRH neurons. We examined the potential ability of IGF-1 to regulate axonal outgrowth of GHRH neurons, and how it is controlled by nutrition using *in vitro* arcuate explant cultures.

## Materials & Methods

### Animal experiments

In order to measure axon growth of GHRH neurons *in vitro*, we used the GHRH-eGFP C57Bl/6J mouse model generated by I.C. Robinson and colleagues [21]. This mouse model was built with an eGFP DNA sequence merged below the signal peptide of the human growth hormone in order to drive the eGFP protein in secretory vesicles. This construct was included in the first intron of the GHRH sequence before micro-injection into fertilized mouse oocytes. We used the mouse line 39 in which the eGFP expression is not too strong in the hypothalamus and without ectopic eGFP expression nor any alteration of the GHRH gene expression [21]. This mouse model has been previously used by various laboratories [21,22,23,24,25,26]. Mice were housed under standard SOPF conditions in individually ventilated cages at 22°C with a 12 h light/dark cycle and free access to water and a commercial rodent diet. Heterozygous GHRH-eGFP males (GHRH-eGFP^+/0^) maintained under a C57Bl/6J genetic background were bred with wild type C57Bl/6J females. Pregnant dams were isolated and housed individually to ensure that pups nutrition during lactation was provided by only one dam. Nutritional restriction during lactation was achieved for the entire litter by cross-fostering pups at birth: “normally fed” pups were obtained from a litter size of six pups per dam and “underfed” pups from a litter size of 10 pups per dam, as described previously [6,27]. Male and female pups were homogenously balanced in each litter and individually identified at 4 days of age by toe clipping. At 28 days of age, all animals were randomly weaned at 6 per cage, independently of their previous diet. From this day, all mice were fed *ad libitum* with a standard commercial diet. For body growth measurement, pups body weight was followed from 10 to 90 days of age. Mice were used at 7, 10, 20 and 90 days of age, depending of the experiment. Tissues and blood sample were harvested from 10, 20 and 90 days-old mice under deep isoflurane anaesthesia. Brain dedicated to explants cultures were harvested from seven days-old pups by quick decapitation. The number of mice/litters used is indicated in results and legends. For experimentations and statistical analyses, all pups originate from at least three different litters to avoid any maternal bias. All animal procedures were performed in accordance with institutional directives for the care of laboratory animals and approved by the French national ethics committee 05 Charles Darwin (Project protocol agreement number Ce5/2012/006).

### In Situ Hybridization and Immunohistochemistry

For *in situ* hybridization, 16 μm frozen sections of brain from 10 day-old male mice were prepared as described previously [21]. Briefly, brain slices were fixed in 4% paraformaldehyde (PFA) and hybridized with a digoxigenin-labeled (Roche) GHRH-RNA probe [21]. Hybridized probe was detected with an alkaline phosphatase-conjugated anti-Dig antibody (Table 1) and NBT/BCIP (Promega) coloration. For brain GHRH immunohistochemistry, the entire median eminence from paraffin embedded brains from normally fed or underfed P10 male pups was sliced (7 μm thickness), and one of every ten slices were mounted on slides for the subsequent innervation analysis. The area of innervation for each slice was determined and the global ME innervation estimated for each individual (n = 3 normal and 4 underfed). After deparaffinization and rehydration, the slices were subjected to heat-induced antigen retrieval in 10 mM Citrate buffer, pH6. The slices were then incubated with anti-GHRH and anti-Panendothelial Cell Antigen MECA-32 antibodies (Table 1). Sequential incubation with secondary antibodies and revelation reagents were then performed: first, slices were incubated with a biotinylated anti-rabbit secondary antibody (Table 1), followed by amplification with the ABC Elite kit (Vector) and revealed using DAB (Dako) for the GHRH IHC; secondly, slices were then incubated with biotinylated anti-rat secondary antibody (Table 1), followed by amplification with the ABC Elite kit (Vector) and revealed using VIP (Vector) for the MECA-32 IHC. The slices were mounted and visualized with an Olympus BX43 with DP73 camera. For the growth hormone (GH) and prolactin (PRL) immunohistochemistry, pituitary glands from 20 day-old male pups were fixed in 4% PFA and embedded in paraffin. The choice of 20 days of age for pituitary analysis was based on the dynamics of pituitary development, as previously shown [6,7]. Pituitary glands were sliced (7μm thickness) and were mounted on slides (three series in parallel). After deparaffinization and rehydration, the first series was incubated with a rabbit anti-GH antibody and the second one with a rabbit anti-PRL antibody (see Table 1), followed by incubation with Dylight-549 donkey anti-rabbit IgG for both (Table 1). The density of somatotrophs cells was measured with the area of GH labeling in three identical fields (250x250μm) per picture, with 5–11 pictures per individual (n = 3 normal and 4 underfed) acquired with fluorescent microscope under identical conditions. The same approach was performed for lactotrophs cells (PRL) density measurement. All slides were mounted and then visualized with an Olympus BX612 fluorescence microscope and DP71 CCD camera under identical acquisition conditions.

**Table 1:** Primary and secondary antibodies.

Peptide/Protein Target	Name of Antibody	Manufacturer, Catalog Number, or Name of Source	Species Raised in Monoclonal or Polyclonal	Dilution Used
AgRP	Agouti-Related Protein (AGRP) (82–131) Amide (Mouse) Antibody	Phoenix Pharmaceuticals, H-003-57	Rabbit, polyclonal	1/ 500
Akt	Akt (pan) (C67E7) Rabbit mAb (HRP Conjugate)	Cell Signaling Technology, #8596	Rabbit, monoclonal	1/ 2000
Chicken IgY	Goat Anti-Chicken IgY H&L (DyLight 594)	Abcam, ab96949	Goat, polyclonal	1/ 400
Digoxigenin	Anti-Digoxigenin-AP, Fab fragments	ROCHE, 11093274910	Sheep, polyclonal	1/ 2000
Erk1/2	p44/42 MAPK (Erk1/2) (137F5) Rabbi mAb	Cell Signaling Technology, #4695	Rabbit, monoclonal	1/ 1000
GFP	Anti-GFP antibody	Abcam, ab6556	Rabbit, polyclonal	1/1000
GH	Rabbit antiserum to rat growth hormone for immunocytochemistry	National institute of diabetes & digestive & kidney diseases (NIDDK), AFP5641801	Rabbit, polyclonal	1/20000
GHRH	Anti-VC-15	Proteogenix, home made	Rabbit, polyclonal	1/1000
IGF-1R	IGF-1 Receptor β Antibody	Cell Signaling Technology, #3027	Rabbit, polyclonal	1/ 1000
MECA-32	Purified Rat Anti-Mouse Panendothelial Cell Antigen MECA-32	BD Pharmingen, 553849	Rat, monoclonal	1/500
Mek1	Polyclonal Anti- Dual specificity mitogen-activated protein kinase kinase 1, MAP2K1	Boster Biological Technology, PA1376	Rabbit, polyclonal	1/ 3000
Neurofilament	Anti-160 kD Neurofilament Medium anibody	Abcam, ab72998	Chicken, polyclonal	1/1000
Phospho Akt	Phospho-Akt (Ser473) (D9E) XP Rabbit mAb	Cell Signaling Technology, #4060	Rabbit, monoclonal	1/ 2000
Phospho Erk1/2	Phospho-p44/42 MAPK (Erk1/2) (Thr202/Tyr204) (D13.14.4E) XP Rabbit mAb	Cell Signaling Technology, #4370	Rabbit, monoclonal	1/ 2000
Phospho IGF-1R	Phospho-IGF-I Receptor β (Tyr1135/1136)/Insulin Receptor β (Tyr1150/1151) (19H7) Rabbit mAb	Cell Signaling Technology, #3024	Rabbit, monoclonal	1/ 500
Phospho Mek1	Anti-Mek1 (phospho S298) antibody [EPR3338]	Abcam, ab96379	Rabbit, monoclonal	1/ 3000
PRL	Rabbit antiserum to rat Prolactin for immunocytochemistry	National institute of diabetes & digestive & kidney diseases (NIDDK), AFP5641801	Rabbit, polyclonal	1/40000
Rabbit IgG	Biotinylated anti-rabbit IgG (H+L)	Vector Laboratories, BA-1000	Goat, polyclonal	1/200
Rabbit IgG	Donkey Anti-Rabbit IgG H&L (DyLight 550) preadsorbed	Abcam, ab98499	Donkey, polyclonal	1/ 400
Rabbit IgG	Goat Anti-Rabbit IgG H&L (DyLight 488)	Abcam, ab96883	Goat, polyclonal	1/ 400
Rabbit IgG	Anti-Rabbit IgG (whole molecule)-Peroxidase	Sigma-Aldrich, A0545	Goat, polyclonal	1/ 20000
Rat IgG	Biotinylated anti-rat IgG (H+L)	Vector Laboratories, BA-4001	Rabbit, polyclonal	1/200

List of primaries and secondaries antibodies used for immunohistochemistry and western blot experiments with manufacturers’ references and dilutions used.

### Arcuate explant culture experiments

For explants culture experiments, brain was harvested from 7 days old normally or underfed pups after rapid decapitation. Since a minimum amount of explants is required for the quality of explants cultures, brain from both males and females were pooled before to be processed all together. Thus, although females are less sensitive to nutritional restriction than males, which may dampen the effects observed, arcuate nucleus explants were harvested from pups of both sexes to obtain a sufficient amount of material from each litter. Brain expression of eGFP was directly verified at the median eminence level with inverted fluorescent microscope (Evos Cell Imaging, Evos). Organotypic slices were prepared and then cultured for 48 h as described previously [28]. Briefly, 300 μm brain slices were incubated on culture membranes (Whatmann) in MEM Medium (Life technologies) supplemented with 10% FCS, 0.5% glucose, and 1% penicillin/streptomycin. Arcuate nuclei were then microdissected and cultured in neurobasal medium (Life technologies) supplemented with methylcellulose, B-27 supplement (B-27, Life Technologies), glucose, L-glutamine, and penicillin/streptomycin (Life technologies). Of note, the B27 supplement contains insulin for neuron survival at a final concentration of 58 nM, according to our determinations (Access Ultrasensitive Insulin kit #33410 on an Access 2 automate, Beckman-Coulter). Cultures were performed in four wells plates, allowing one control and three treated conditions for each experiment. Each well was filled with eight explants. One plate could be filled from one normally fed litter and two plates could be filled from one underfed litter. Explants experiments with normally fed or underfed pups were conducted sequentially, depending the production of mice. After 24 h in culture, the explants were treated with 100 ng/ml IGF-1 (13.2 nM, R&D systems) alone or in combination with 1 μM OSI-906 (Linsitinib, Selleckchem) for another 24 h. The explants were then fixed for 30 min in 4% PFA and subjected to immunohistochemistry using primary antibodies directed against neurofilament, GFP, AgRP, IGF-IR, and their corresponding secondary antibodies (See Table 1). Neurofilaments and AgRPs were visualized under a 4X objective, and GFP under a 10X objective since these axons were smaller, using an Olympus BX612 fluorescence microscope and DP71 CCD camera.

For the analysis of axon length, the NeuronJ plugin of ImageJ software was used, as previously described [29]. Briefly, the length of a maximum of 40 axons (mean 24.1 ± 1.5 axons) for both normal and underfed was measured for each explants. The global axon growth for each condition was calculated as the mean of the eight quantified explants. The global axon growth for the three treated conditions were normalized against their respective controls (basal condition) for each plate due to the variability of the explant cultures. Since pups were pooled for the explants culture, each plate was considered as one single experiment (n = 1) for statistical analyses, and data are presented as the mean of relative axon growth calculated from various experiments (n = 6–9 experiment depending of the test, the number is given in results and legends). Globally, a total of 14 litters of normally fed and 20 litters of underfed pups were used for explants cultures.

### Biochemical analysis

Plasma IGF-1 levels were determined from 10 and 90 days old mice using the Mouse/Rat IGF-I (Mediagnost) ELISA kit according to the manufacturer’s instructions. IGF-I/IGFBP complexes are dissociated during this assay and the IGFBPs are saturated with IGF-2, thus allowing the measurement of total circulating IGF-I (spectrophotometer TECAN, GENios Pro). For Western blot analysis, micro-dissected arcuate nuclei from one litter of 7 day-old pups (both sexes) normally fed or underfed (n = 4–6 litters per group) were recovered in neurobasal medium supplemented with B-27 minus insulin, for 2 h in a separate set of experiments. Arcuate nuclei explants were stimulated with IGF-1 (100ng/ml) or control medium for 15 min. Proteins of stimulated and un-stimulated explants were then extracted as previously described [30]. For the cytosolic fraction, samples were homogenized in a hypertonic buffer consisting of 10 mM HEPES pH 7.9, 10 mM KCl, 0.1 mM EDTA, and 0.1 mM EGTA, containing a cocktail of both protease and phosphatase inhibitors (Roche). After 15 min of incubation on ice, 6.3μl of 1%NP-40 per 100 μl buffers was added, the samples vortexed briefly, and centrifuged at 5000xg for 1 min at 4°C. The resulting supernatant, enriched for the cytosolic fraction, was transferred to a new tube for analysis by Western blot. The pellet was resuspended in a second buffer consisting of 20 mM HEPES pH 7.9, 0.4 M NaCl, 1 mM EDTA, and 1 mM EGTA. The suspension was shaken for 15 min at 4°C and centrifuged at 13000xg for 5 min at 4°C. The pellet, enriched in membranous proteins, was resuspended in 150μL PBS. The cytosolic fraction was concentrated using the Centrifugal Filter Units Amicon Ultra kit (Millipore). The protein concentration of all fractions was then determined by nanodrop. For each sample, 10 μg of cytoplasmic proteins (AKT, MEK, ERK) or 7 μg of membrane proteins (IGF-1R) were separated on NuPAGE Bis-Tris (4–12%) gels (Life technologies) and transferred to PVDF membranes. The membranes were then incubated with primary antibodies against phospho-ERK1/2 (p-ERK1/2), ERK1/2, phospho-MAP2K1 (p-MEK1), MAP2K1 (MEK1), phospho-AKT (p-AKT), AKT, Actin, phospho-IGF-1R (p-IGF-1R), and IGF-1R, using an anti-rabbit HRP-IgG as a secondary antibody (Table 1). The membranes were then revealed with SuperSignal West Pico or Femto Chemiluminescent Substrate (37080, 34095, Thermo Scientific) using a high sensitivity, cooled charge-coupled device (CCD) 6 Mpx camera on a ChemiDoc Touch Imaging System (Biorad).

### Measurement of gene expression

Gene expression was determined as described previously [6,7]. Briefly, total RNA from the pituitary gland of adult male mice was extracted using the silica membrane method according to the manufacturer’s instructions (NucleoSpin RNA II, Macherey-Nagel). Total RNA from the pituitary gland of 10-day-old mice was extracted using the phenol-chloroform method (RNAble, Eurobio). This method was used for 10 days-old pups’ pituitary since silica membranes method does not allow RNA isolation from such small quantities. A total of 1 μg of RNA was reverse transcribed using random hexamers, RNAse inhibitor, and RevertAid H Minus Reverse Transcriptase (Fermentas). Real time PCR was performed in duplicate with 6 ng of cDNA on an Applied Biosystem 7300 PCR System. The primers used were GH (Fwd TCACTGCTTGGCAATGGCTA, Rv ACAGACTGGACAAGGGCATG), and Pit-1 (Fwd CACGGCTCAGAATTCAGTCA, Rv CTGATGGTTGTCCTCCGTTT). The abundance of mRNA was measured using the standard curve method and normalized against the housekeeping gene H3f3b (Fwd CCAGAAATCGACTGAGCTGCTCAT, Rv GCTGCACTTTGAAACCTCAAGTCG).

### Statistical analysis

All data are presented as the mean ± SEM. Comparisons between two groups were performed using the non-parametric test Mann Whitney and the growth curve was analyzed by repeated measures Two-way ANOVA. For explant cultures, results from dual immunohistochemistry (IHC) experiments (e.g. NF/GFP) were analyzed by two-way ANOVA with Bonferroni correction for multiple comparisons, and the results of singular IHC (AgRP) were analyzed by one-way ANOVA with Newman Keuls’ multiple comparison test. Statistically non significant results were abbreviated “NS” in text. Statistically significant results are marked with asterisks, * *p* < 0.05; ** *p* < 0.01; *** *p* < 0.001.

## Results

### Underfeeding during lactation results in permanent growth delay and reduced secretion of growth hormone

**Fig 1:**
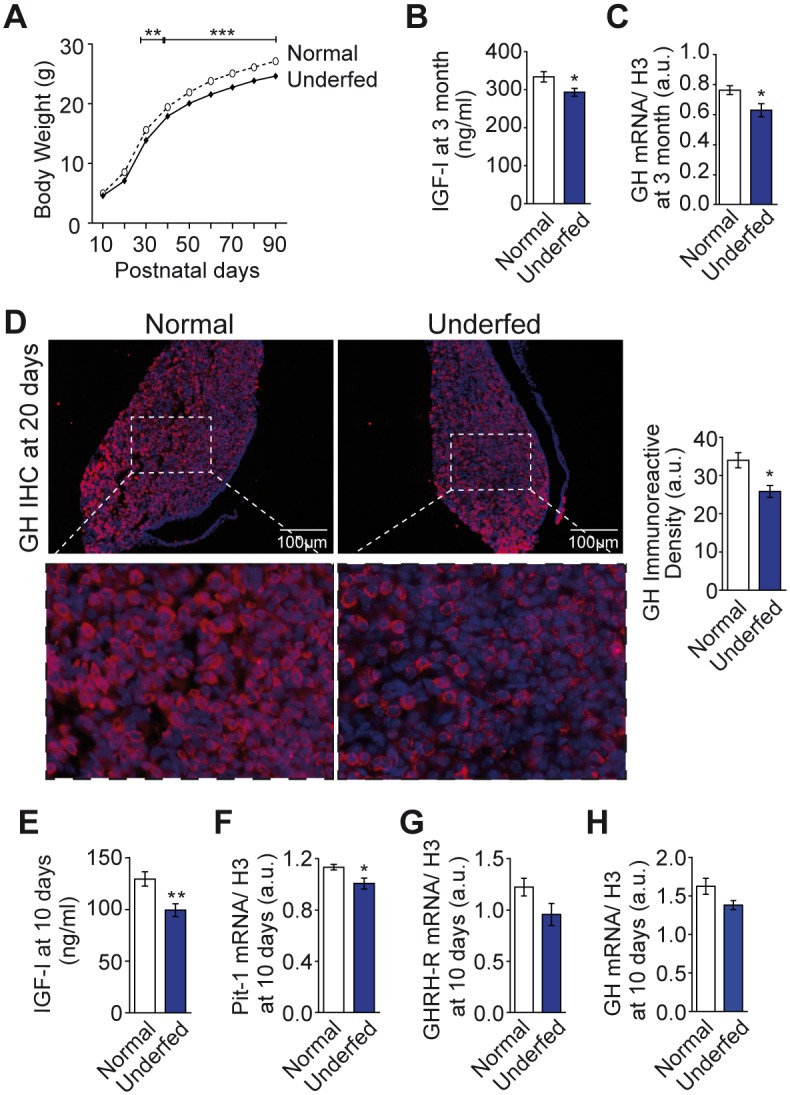
Underfeeding during the early postnatal period induces a permanent growth delay. Increasing litter size from 6 (Normally fed) to 10 (Underfed) pups per dam permanently delayed postnatal growth of male pups, as observed with postnatal body weight gain (n = 23–25 per group) that persisted into adulthood (**A**). This was associated with low circulating plasma levels of IGF-I in 3 month-old male mice previously underfed during lactation (n = 7–9 per group) (**B**), accompanied by decreased levels of GH mRNA in the pituitary gland (n = 9 per group) (**C**). Representative micrograph of GH-producing somatotroph cells (labeled in red by immunohistochemistry, counterstaining with DAPI) from normally fed and underfed 20 day-old male pups (**D**, left and middle panels respectively) suggest a somatotrophs pituitary hypoplasia (**D**, right panel) (n = 3–4 per group). This was preceded by lower plasma levels of IGF-1 (n = 8 per group) (**E**) and decreased expression of the somatotroph differentiation factor, Pit-1 (n = 5 per group) (**F**) in 10 day-old underfed male pups. Moreover, this was associated with a non—significant tendency of decreased expression levels of GHRH receptor (GHRH-R) (**G**) (n = 5 per group), and of GH (**H**) (n = 5–7 per group) in 10 day-old underfed male pups. All data are presented as the mean ± SEM. Gene expression determinations are normalized against the histone H3 gene (**C, F**). Comparisons were performed by repeated measure two-way ANOVA analysis (**A**) or Mann Whitney analysis (**B**–**F**), with * p < 0.05 and *** p < 0.001.

GHRH-eGFP^+/0^ C57Bl6/J males were mated with wild type C57Bl6/J females. We restricted nutrition during lactation (P1–P16) in a modest and transient manner [Kappeler, 2009 #9] by increasing the litter size of C57Bl6/J dams the day of birth from six (“normal”) to 10 (“underfed”) pups. Normal and underfed pups were raised in parallel and the effects of this nutritional restriction on body growth were assessed. Males from underfed (large) litters showed a delay of postnatal growth that persisted into adulthood (Fig 1A). Females littermates exhibit a lighter impact of under-nutrition and were not studied further (see S1 Fig). Three month-old male adult mice subjected to nutritional restriction during lactation had lower circulating IGF-I plasma levels than mice from normal litters (Fig 1B). This was associated with lower pituitary growth hormone (GH) mRNA levels (Fig 1C) and permanent pituitary hypoplasia of GH-secreting somatotroph cells from 20 days of age (Fig 1D), as we previously showed in adults [6,7]. These modifications are associated with a non significant decrease of GHRH receptor (GHRH-R) and Pit-1 gene expression in 3 month-old male mice (S2 Fig). The somatotrophs hypoplasia seems specific since lactotroph cells that originate from the same precursor as somatotrophs ones do not present any decrease in same animals and even tend to be increased (non significant, see S3 Fig).

The low activity of the somatotropic axis in pups from underfed litters suggests alterations in hypothalamus-pituitary development during the early postnatal period [6,7]. In agreement, underfed 10 day-old male pups displayed decreased circulating levels of IGF-I (Fig 1E) and lower body weight (Normal: 4.98 ± 0.16 *vs* Underfed: 4.59 ± 0.08; n = 23–24; t-test: *p* < 0.05), as well as lower expression of the somatotroph-differentiating factor Pit-1 in the pituitary gland (Fig 1F). This was associated with a non-significant decrease of GHRH-R (*p* = 0.17; Fig 1G) and of GH (*p* = 0.07; Fig 1H) expression levels. The decrease of Pit-1 in the pituitary glands of 10 day-old underfed pups suggests a lack of GHRH stimulation, the latter of which has been shown to stimulate somatotroph cell proliferation through Pit-1 [8]. These findings suggest that reprogramming of the somatotropic axis induced by nutritional restriction may involve GHRH neurons which are still developing during this early postnatal period.

### Postnatal underfeeding delays innervation of the median eminence by GHRH neurons

**Fig 2:**
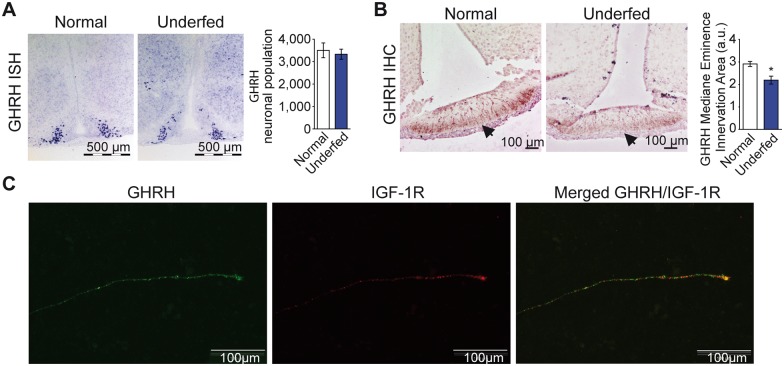
The development of GHRH neurons is altered in response to underfeeding. The numbers of GHRH neurons in the arcuate nucleus of the hypothalamus was estimated by *in situ* hybridization in 10 day-old normally fed or underfed male pups (n = 3 per group) (**A**). Concomitantly, the area of innervation of the median eminence (highlighted with the arrow) by axons of GHRH neurons was lower in 10 day-old underfed male pups (n = 4 per group) (**B**). Illustrative immunohistochemistry from *in vitro* cultured arcuate nucleus explants from 7 day-old normally fed pups show that IGF-1R (in red, middle panel) is preferentially enriched in the distal part of growing GHRH axon (in green, left panel). Merged picture is in the right panel (**C**). Data are presented as the mean ± SEM. All Comparisons were performed using Mann Whitney analysis, with *: p < 0.05.

We first determined whether low IGF-I levels in underfed mice were associated with a loss of GHRH neurons, as IGF-I is very well known for its proliferative and anti-apoptotic actions. The number of GHRH neurons in normally fed and underfed 10 day-old male pups was similar by *in situ* hybridization with a GHRH antisense mRNA probe (Fig 2A), suggesting that food restriction does not alter the ontogeny of GHRH neurons. We thus looked for morphological modifications of developing GHRH neurons. We observed a lower anti-GHRH immunoreactive area in the median eminence (the target of GHRH neurons) of 10 day-old underfed male mice than in age-matched normally fed male mice (Fig 2B). This decrease disappeared by 20 days of age as previously shown [7]. These results suggest that innervation of the median eminence by GHRH neurons may be delayed by under-nutrition, probably due to an alteration of their axonal elongation. This may explain the lower Pit-1 expression levels observed in pituitary glands in 10 day-old underfed pups.

Data from IHC experiments in brain suggest that GHRH neurons of the arcuate nucleus of the hypothalamus innervate the adjacent median eminence by forming a dorso-lateral loop that reaches the median eminence from the posterior part of the brain, making their axons very difficult to follow *in vivo* (E.M. & L.K., personal communication). We thus applied an *ex vivo* approach on isolated and *in vitro* cultivated explants of the arcuate nucleus from normally fed mice to further examine the role of IGF-I in the elongation of axons of these hypothalamic neurons [28,31]. IGF-I is virtually expressed by all cells and the immunohistochemistry of arcuate explants isolated from the brains of normally fed GHRH-eGFP pups showed that the IGF-1R seem to be concentrated in the distal part of growing GHRH axons (Fig 2C), suggesting that IGF-I may regulate axonal elongation in arcuate neurons.

### IGF-I preferentially stimulates axon growth in GHRH arcuate neurons

Arcuate nucleus explants were micro-dissected from 7 day-old GHRH-eGFP pups of both sexes. Since a minimum amount of explants is required for the quality of explants cultures, brain from both males and females were pooled before to be processed all together. Thus, although females are less sensitive to nutritional restriction than males, which may dampen the effects observed, arcuate nucleus explants were harvested from pups of both sexes to obtain a sufficient amount of material from each litter. In unstimulated (Control condition) explant cultures, the axons of GHRH neurons (labeled with eGFP IHC) were shorter than those of total arcuate neurons labeled with neurofilament (NF) (Control-GHRH: 420 ± 41 μm *vs*. Control-NF: 600 ± 58 μm, n = 5 and 6 experiments per group, respectively, *p* < 0.05).

**Fig 3:**
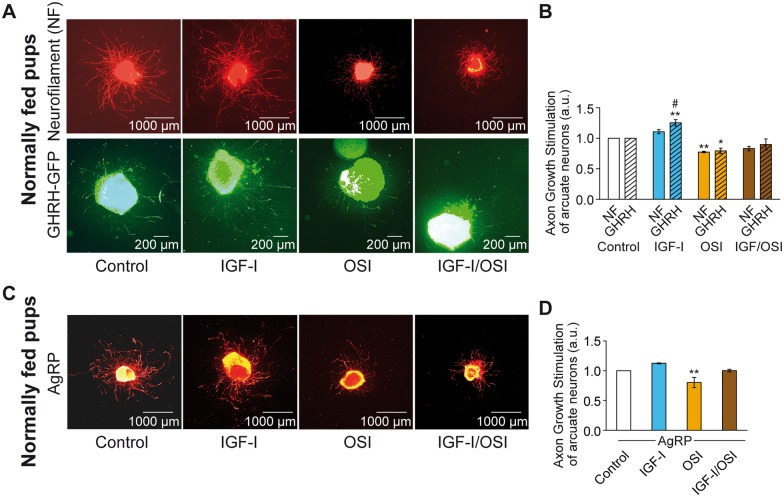
IGF-I stimulates axon growth of GHRH neurons in arcuate nucleus explants. Illustrative micrograph of *in vitro* cultured explants of arcuate nuclei of hypothalamus micro-dissected from 7 day-old normally fed GHRH-eGFP^+^ pups of both sexes are shown (**A**) in basal condition, under IGF-I stimulation or in presence of OSI-906 inhibitor (alone or in combination). Axons from whole arcuate (NF) and GHRH neurons are labeled by a dual-IHC for neurofilament (NF, top panels in red) and eGFP (GHRH-eGFP, lower panels in green), respectively. Quantification of relative axon growth stimulated by IGF-I and/or its inhibitor OSI-906 (OSI) in 24 h for NF (plain bars) and GHRH-eGFP (GHRH, dashed bars) are presented (**B**). In a separated set of experiment, a similar experiment was performed on NPY/AgRP neurons labeled by IHC for AgRP (in orange) (**C**). The relative axon growth of this other subpopulation of arcuate neurons under IGF-I stimulation and/or its inhibitor OSI-906 (OSI) is presented (**D**). Data are presented as the mean ± SEM calculated from n = 5–6 (**B**) and n = 4 (**D**) experiments per group, respectively (see results section for details). Results were compared using a two-way ANOVA analysis with the Bonferonni post-test (**B**) or one-way ANOVA analysis with the Newman Keuls post-test (**C**). *: p < 0.05 and **: p < 0.01 for each treatment *vs*. Control; #: p < 0.05 for GHRH *vs*. NF.

In explant cultures treated with IGF-I (13.2 nM) for 24 h, axon growth in NF-labeled arcuate neurons was not significantly modified (IGF-I-NF: 662 ± 61 μm *vs*. Control-NF: 600 ± 58 μm, n = 6 experiments per group, 1.11 ± 0.04 fold, Non Significant: NS; Fig 3A and 3B). In contrast, axonal growth of GHRH neurons in the same explants was significantly stimulated by IGF-I (IGF-I-GHRH: 498±68 μm *vs*. Control-GHRH: 420±41 μm, n = 5 experiments per group, 1.25 ± 0.06 fold, *p* < 0.01; Fig 3A and 3B). We used the IGF-1R inhibitor OSI-906 (*Linsitinib*) [32] alone, or in combination with IGF-I, to confirm that axon growth was specifically stimulated by IGF-I. Indeed, inhibition of the IGF-1R impaired axon growth in GHRH (IGF-I/OSI-GHRH: 0.9 ± 0.10 fold *vs*. IGF-I-GHRH: 1.25 ± 0.06 fold, n = 4–5 experiments per group, *p* < 0.0001; Fig 3B), but curiously also in NF neurons (IGF-I/OSI-NF: 0.83 ± 0.04 fold *vs*. IGF-I-NF: 1.16 ± 0.02 fold, n = 5 experiments per group, *p* < 0.0001). Similar results were obtained using a second IGF-1R inhibitor, *picropodophyllotoxin* (PPP; data not shown). The diminution of axonal growth in NF^+^ neurons by OSI may be due to the requirement of IGF-I and insulin signaling for neuron survival (insulin is a crucial component of the B27 supplement of the cell culture media for neuron survival, measured at 58 nM), which also associates with the positive basal axon growth.

The fact that IGF-I significantly stimulated axon growth in GHRH, whereas it did not in NF^+^ axons, suggests a cell-selective effect. This was further investigated in another subpopulation of arcuate neurons, the orexigenic NPY/AgRP neurons that have been previously described to respond to the stimulating effect of leptin during the same period of development [33,34]. NPY/AgRP neurons were labeled by IHC in a separated set of experiments, using arcuate explants culture harvested from 7 day-old normally fed pups. IGF-I did not significantly stimulate axonal growth of AgRP neurons (AgRP-IGF-I: 559 ± 35μm *vs*. AgRP-Control: 497 ± 34μm; 1.13 ± 0.01 fold, n = 4 experiments per group, NS; Fig 3C and 3D) although OSI906 did inhibit it (Fig 3D). Here again, the diminution of axonal growth in AgRP neurons by OSI may be due to the inhibition of IGF-I and insulin signaling, which may impact the neurons survival and their basal axon growth. Indeed, insulin is a crucial component of the B27 supplement for neuron survival. Altogether, these results suggest that IGF-I preferentially stimulates axon elongation of GHRH neurons of the arcuate nucleus from normally fed pups (IGF-I-GHRH *vs*. IGF-I-NF: *p* < 0.01).

### Underfeeding alters the response of GHRH neurons to IGF-I

The same experiments as those just described for normally fed pups were performed with arcuate nucleus explants isolated from 7 day-old underfed GHRH-eGFP pups of both sexes to further investigate whether axon elongation of GHRH neurons stimulated by IGF-I may be affected by underfeeding.

*In vitro* basal axon growth (Control condition) of whole arcuate neurons (NF^+^) originating from underfed pups was similar to the growth of those isolated from age-matched normally fed pups (NF-underfed: 553 ± 26 μm *vs*. NF-Normally fed: 600 ± 58 μm, n = 9 and 6 experiments per group, respectively, Non Significant: NS). However, *in vitro* basal axon growth of GHRH neurons from underfed pups was lower than the growth of GHRH neurons isolated from normally fed pups (GHRH-underfed: 296 ± 36 μm *vs*. GHRH-Normally fed: 420 ± 41 μm, n = 7 and 5 experiments per group, respectively, p < 0.05).

**Fig 4:**
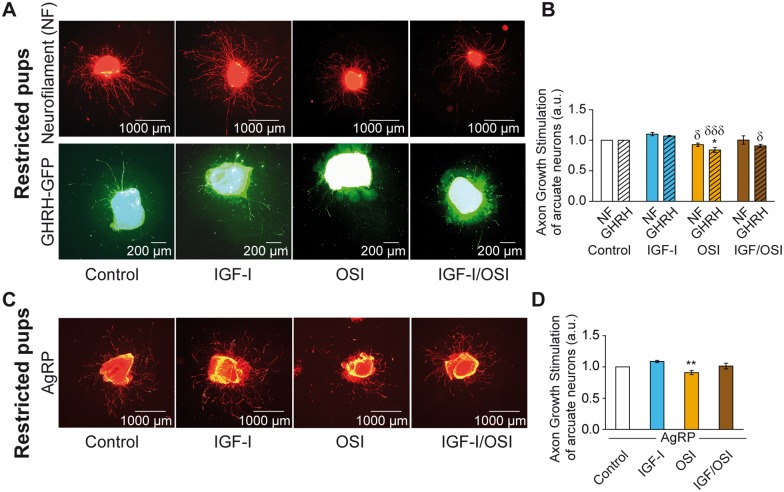
GHRH neurons from underfed pups do not respond to IGF-I stimulation. Illustrative micrograph of *in vitro* cultured explants of arcuate nuclei of hypothalamus micro-dissected from 7 day-old underfed GHRH-eGFP^+^ pups of both sexes are shown (**A**) in basal condition, under IGF-I stimulation or in presence of OSI-906 inhibitor (alone or in combination, with axons from whole arcuate (NF) and GHRH neurons labeled by a dual-IHC for neurofilament (NF, top panels in red) and eGFP (GHRH-eGFP, lower panels in green), respectively. Quantification of relative axon growth stimulated by IGF-I and/or its inhibitor OSI-906 (OSI) in 24 h for NF (plain bars) and GHRH-eGFP (GHRH, dashed bars) are presented (**B**). In a separated set of experiment, a similar experiment was performed on NPY/AgRP neurons labeled by IHC for AgRP (in orange) (**C**). The relative axon growth of this other subpopulation of arcuate neurons under IGF-I stimulation and/or its inhibitor OSI-906 (OSI) is presented (**D**). Data are presented as the mean ± SEM calculated from n = 4 (**B**) and 5 (**D**) experiments per group (see results section for details). Results were compared using a two-way ANOVA analysis with the Bonferonni post-test (**B**) or one-way ANOVA analysis with the Newman Keuls post-test (**C**). *: p < 0.05 and **: p < 0.01 for each treatment *vs*. Control; δ: *p* < 0.05, and δδδ: *p* < 0.001 for each treatment *vs*. IGF-I.

Stimulation of explants from underfed GHRH-eGFP pups with IGF-I did not significantly induce axon growth in NF^+^ arcuate neurons (NF-IGF-I *vs*. NF-Control: 1.10 ± 0.03 fold, n = 4 experiments per group, NS; Fig 4A and 4B), similar to the results for normally fed pups. In the same way, AgRP neurons from underfed pups also did not respond to IGF-I (AgRP-IGF-I *vs*. AgRP-Control: 1.09 ± 0.02 fold, n = 5 experiments per group, NS; Fig 4C and 4D). However, GHRH neurons harvested from underfed GHRH-eGFP pups did not respond to IGF-I stimulation (GHRH-IGF-I *vs*. GHRH-Control: 1.07 ± 0.01 fold, n = 4 experiments per group, NS; Fig 4A and 4B), in contrast to normally fed pups (Fig 3A and 3B), and were only moderately sensitive to the inhibition of the IGF-1R by OSI (GHRH-OSI *vs*. GHRH-Control: 0.84 ± 0.04 fold, n = 4 experiments per group, p < 0.05; Fig 4B). This moderate effect of OSI is probably due to its supplementary inhibitory effect on the insulin signaling pathway that is crucial for neuron survival, as explained above in experiments on normally fed mice. These data suggest that GHRH neurons from underfed pups are insensitive to IGF-I despite normal culture conditions.

### Underfeeding alters the IGF-I signaling pathway

**Fig 5:**
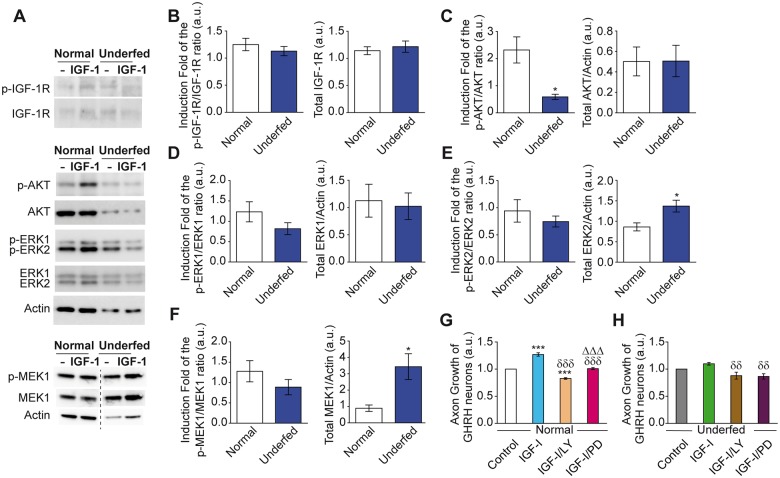
Alterations of the IGF-IR signaling pathways in arcuate explants from underfed pups. Activation of key elements of IGF-1R signaling pathways were measured by Western blot analysis as illustrated with representative blots in basal condition (-) or 15 min after IGF-I stimulation (IGF-I) (**A**). Quantification indicates activation of the IGF-1R (fold induction of the p-IGF-1R/ IGF-1R ratio) by IGF-I stimulation relative to basal levels (left panel), and total IGF-1R protein levels (right panel) in arcuate explants harvested from normally fed and underfed pups of both sexes (**B**). Similarly, activation of AKT (fold induction of the pAKT/AKT ratio) (**C**), ERK1 (**D**), ERK2 (**E**) and MEK-1 (**F**) are presented (left panels) with their respective total protein levels (right panels) (n = 4–6 per group). *In vitro* explant cultures indicate that specific inhibition of PI3K by LY294002 (LY) and of MEK by PD0325901 (PD) impaired axon growth of GHRH neurons in normally fed (n = 4 per group) (**G**) and underfed GHRH-eGFP^+^ pups (n = 6 per group) (**H**). All data are presented as the mean ± SEM with Mann Whitney analysis for Western blot analysis with *: *p* < 0.05 (**V-F**); and one way-ANOVA analysis with the Newman Keuls post-test (**G**, **H**) with *: *p* < 0.05 and ***: *p* < 0.001 for each treatment *vs*. Control; δδ: *p* < 0.01 and δδδ: *p* < 0.001 for each treatment *vs*. IGF-I; Δ Δ Δ: *p* < 0.001 IGF-I/PD *vs*. IGF-I/LY.

The main elements of the IGF-1R signaling pathway were measured by Western blot analysis, despite the highly limited amount of protein that could be harvested from micro-dissected arcuate nuclei from normally fed or underfed 7 day-old pups. As with explants cultures, micro-dissected arcuate nuclei were harvested from both sexes. Overall, underfed pups had similar IGF-1R protein levels in their arcuate nucleus as age-matched normally fed pups (Fig 5A and 5B right panel). In addition, a similar induction of IGF-1R activation (p-IGF-1R/IGF-1R ratio) was observed after 15 min of IGF-I stimulation in both underfed and normally fed pups relative to the basal condition (Fig 5A and 5B, left panel). These data suggest that both total IGF-1R levels and its activation were not altered by nutritional restriction.

We thus determined the activation capacities of the two principal signaling pathways activated by the IGF-1R, the PI3K/AKT, and the ERK/MEK pathways. The induction of activated AKT (increase of the p-AKT/AKT ratio) following IGF-I stimulation was much lower in arcuate nuclei isolated from underfed pups than those from normally fed pups (Fig 5A and 5C, left panel). This impairment appeared to involve the activation of AKT as the level of total AKT protein was similar for the two groups (Fig 5A and 5C, right panel). For the ERK/MEK pathway, the induction of activated ERK-1 (increase of the p-ERK-1/ERK-1 ratio) by IGF-I stimulation was not significantly modified in arcuate nuclei harvested from underfed pups relative to normally fed pups (Fig 5A and 5D left panel, NS: p = 0.126). Total ERK-1 protein levels were also not modified by nutritional restriction (Fig 5A and 5D, right panel). Induction of activated ERK-2 (increase of the p-ERK-2/ERK-2 ratio) following IGF-1 stimulation was also similar for underfed and normally fed pups (Fig 5A and 5E, left panel). However, total ERK-2 protein levels were elevated in underfed pups (Fig 5A and 5E, right panel). We observed similar results for the MEK protein as for the ERK-2 protein: a similar response of the underfed and normally fed pups to IGF-I stimulation (Fig 5A and 5F, left panel), and an elevated level of total protein in underfed pups relative to normally fed pups (Fig 5A and 5F, right panel). Altogether, these data suggest that the absence of axon elongation observed for GHRH neurons in underfed pups is associated with impaired activation of the PI3K/AKT signaling pathway, whereas the activation capacity of the ERK/MEK pathway is maintained.

The role of the PI3K/AKT and ERK/MEK signaling pathways in the regulation of axon outgrowth of GHRH neurons were further investigated in arcuate explants from both normally and underfed pups, using specific IGF-1R signaling pathway inhibitors. Normalization respective to the basal conditions of these two sets of experiments (performed in 4 well plates) required presenting the data on two separate graphs since basal axon growth in explants from underfed pups is lower than that of normally fed pups. In agreement with the previous *in vitro* results, co-stimulation of arcuate explants from normally fed pups with IGF-I and the specific PI3K inhibitor LY294002 (LY) significantly inhibited IGF-1 mediated axon growth of GHRH neurons relative to both basal conditions (GHRH-IGF/LY *vs*. GHRH-Control: 0.83 ± 0.03 fold; n = 4 experiments per group; p < 0.001; Fig 5G) and IGF-I alone (p < 0.001; Fig 5G). Treatment of explants with the specific MEK inhibitor PD0325901 (PD) also significantly impaired axon growth of GHRH neurons in response to IGF-I stimulation (GHRH-IGF/PD: *vs*. GHRH-IGF: p < 0.001; Fig 5G), but not relative to basal conditions (GHRH-IGF/PD *vs*. GHRH-Control: 1.00 ± 0.03 fold; NS; Fig 5G). Thus, the inhibition of the PI3K signaling pathway (LY) impaired IGF-1-mediated axon growth to a greater extent than inhibition of the MEK pathway (PD) (GHRH-IGF/PD *vs*. GHRH-IGF/LY: p < 0.001; Fig 5G). This suggests that IGF-I may stimulate axon growth of GHRH neurons in normally fed pups through the PI3K/AKT and, to a lesser extent, the MEK/ERK signaling pathways.

Axon growth of GHRH neurons in explants from underfed mice was not stimulated by IGF-1 and tended to be impaired by the inhibition of PI3K relative to basal conditions (GHRH-IGF/LY *vs*. GHRH-Control: 0.88 ± 0.07 fold, n = 6 experiments per group; NS; Fig 5H), and IGF-1 treatment (GHRH-IGF/LY *vs*. GHRH-IGF: p < 0.01; Fig 5H). A similar pattern was observed for inhibition of the MEK signaling pathway (GHRH-IGF/PD *vs*. GHRH-Control: 0.83 ± 0.06 fold, n = 6 experiments per group; NS; Fig 5H). In contrast to normally fed mice, neurons from underfed pups seemed to be equally sensitive to the inhibition of both the PI3K/AKT and ERK/MEK signaling pathways (underfed: IGF/LY *vs*. IGF/PD: NS; Fig 5H).

## Discussion

Here, we show that IGF-I may be involved in axon elongation in a specific subpopulation of hypothalamus neurons. Indeed, the present study indicates that i) IGF-I preferentially stimulates axon growth of GHRH neurons in arcuate nucleus explants of normally fed pups during lactation; ii) IGF-1 stimulation involves the two main signaling pathways PI3K/AKT and ERK/MEK, with greater involvement of the PI3K/AKT pathway; and iii) GHRH neurons harvested from underfed pups during lactation and cultivated *in vitro* grow less and become insensitive to the growth promoting effect of IGF-I. This loss of response does not involve an alteration of the IGF-1R or ERK/MEK pathway, but is associated with a defect in AKT activation. Finally, iv) nutritional restriction during lactation, which is associated with permanent growth retardation and later metabolic alterations, is associated with lower IGF-I plasma levels and a change of the body growth trajectory. It is preceded by delayed axon elongation of GHRH neurons during the first 10 days of life, a critical developmental window. These physiological outcomes are in agreement with the tight control of IGF-I levels by nutritional supplies.

Prior studies have shown that they are sex and age differences in GHRH neuron development [26]. Our explant experiments were carried out during a period in which there are sex-dependent differences concerning GHRH neurons in the arcuate. Indeed, it was reported that GHRH neurons increase in both sexes during the P1–P10 period, males having more GHRH neurons than females. After P20, the number of GHRH neurons stabilized in males, and continued to increase in females [26]. Since a minimum amount of explants is required for the quality of explants cultures, brain from both males and females were pooled before to be processed. Although females may dampen the effects observed, arcuate nucleus explants were harvested from pups of both sexes to obtain a sufficient amount of material. Our study showed an identical number of GHRH neurons in both normally fed and underfed male pups at P10 (Fig 2A) and highlights the positive effect of IGF-I on GHRH neuron axonal elongation, as shown at P7. The potential sexual dimorphic effect of IGF-I on axon growth of GHRH neurons harvested specifically from male or female pups will require further studies.

IGF-I is well known to improve proliferation and cell survival in virtually all cellular models tested. High IGF-I levels in the brain are associated with increased length and complexity of neuronal dendritic arborization [12,13,14]. It is also strongly associated with axonal determination during the early phase of neuronal differentiation through plasmalemmal expansion [17] induced by the IGF-1Rgc isoform [15,16]. During the later phase of neuronal differentiation and axon elongation, IGF-I induces axonal outgrowth in some neuronal subpopulations. However, this effect is not ubiquitous. Indeed, IGF-1R signaling is associated with axon elongation of corticospinal motor neurons during development [19], but not in adulthood [20]. In contrast, axon regeneration of raphespinal and cerulospinal axons after a spinal cord injury in adults is stimulated by IGF-I [20], despite that corticospinal motor neurons are in close proximity to the raphespinal and cerulospinal neurons [20]. These data suggest that corticospinal motor neurons specifically lose their capacity to respond to IGF-I in adult animals. IGF-1 also does not stimulate axon elongation in the developing lateral olfactory neuron model, but appears to have chemotactic effects [18]. These data suggest that IGF-I may stimulate axon elongation in a cell specific manner and a similar mechanism may operate in the hypothalamus of mice during the early postnatal period. Indeed, IGF-I seems to preferentially stimulate axon elongation of the GHRH neuronal subpopulation, highlighting the complexity of the role of IGF-I in neuronal development.

The PI3K/AKT pathway is strongly implicated in the stimulation of axonal outgrowth in normally fed pups, and underfeeding is associated with impaired AKT stimulation. The specific role of the two IGF-1R signaling pathways has been studied in different models of neuronal cultures. Notably, the stimulation of axonal outgrowth by the PI3K/AKT signaling pathway has been associated with perikaryon-independent plasmalemmal expansion in embryonic pyramidal neurons of the hippocampus [15,17,35]. In contrast, the stimulation of axonal outgrowth in sensory neurons by the ERK/MEK signaling pathway has been associated with both local protein synthesis and the regulation of gene transcription [36,37]. Other studies suggest that both the PI3K/AKT and ERK/MEK signaling pathways present a potential cell-type specific effect. Indeed, the ERK/MEK signaling pathway has been associated with increased axon length in dorsal root ganglion neurons, whereas the PI3K/AKT pathway was associated with axon caliber and branching [38]. Thus, the increase of ERK2 and MEK protein levels in the arcuate of underfed pups may indicate compensation for the altered AKT activation. Further studies will be required to determine the role of the AKT signaling pathway in GHRH axon growth in more details, and particularly if underfeeding is associated with alterations of plasmalemmal expansion of GHRH neurons.

Our present study shows that nutrition plays a crucial role in the development of the somatotropic axis of the hypothalamus-pituitary complex. These data are in agreement with other studies that link nutrition during this critical period and the programming of various hypothalamic neuronal network, notably those of subpopulations that regulate food intake and metabolism in adults, and of the reproductive axis [33,34,39,40]. We previously observed that IGF-1R heterozygous invalidation in the central nervous system specifically leads to a similar postnatal growth defect in male mice [7]. Undernutrition is well known to decrease IGF-I levels [41], and nutritional restriction during the lactating period may alter postnatal growth through a decrease of IGF-I signaling. However, other hormones tightly controlled by nutrition like leptin or insulin cannot be excluded as they were implicated in axonal development of AgRP neurons of the arcuate nucleus [33,40]. Since IGF-1R is enriched in the terminal end of growing GHRH axons, the IGF-1R signaling may be important for their elongation. In this way we observed a stimulating effect of IGF-I on GHRH neurons axon growth preferentially. Unfortunately, IGF-1R is virtually expressed by all cells and IGF-1R quantity cannot be easily measured to explain this preferential effect of IGF-I on GHRH neurons, which remain to be studied in details. Axonal growth of GHRH neurons appears to be a crucial physiological mechanism, controlled by IGF-I, which programs the subsequent production of GH by the pituitary gland, ultimately coordinating organ growth in an endocrine-dependent manner in response to environmental cues during the critical window of the early postnatal period. Growth is indeed a highly integrated process and a tight evaluation of available resources is required to adapt linear growth. The growth programming effect of IGF-I through the regulation of axon growth of GHRH neurons may be one component. This mechanism is thus important from the perspective of the predictive adaptive response hypothesis within the DoHAD, because adult size determines the daily metabolic needs that must be adapted to a predicted environment to optimize survival capacities and reproduction [1,3,4]. Ultimately, this mechanism, accompanied by changes of perinatal IGF-I levels, may partially program ageing and longevity which are also partially controlled in mammals by GH/IGF-I signaling in adulthood [6,7,42,43].

## Supporting Information

S1 Fig Nutritional Restriction does not alter the somatotropic axis in Females. (**A**) Body weight gain during postnatal development is similar between normally fed (n = 17–22) and underfed females (n = 23–25). Coherently, (**B**) plasma IGF-1 circulating levels (n = 10 per group) and (**C**) GH mRNA levels in pituitary (n = 9 per group) at 3month of age are similare between females that have been previously normally fed or underfed during lactation. Moreover, Nutritional restriction is not associated with a decrease of (**D**) plasma IGF-1 circulating levels at 10 days of age (n = 6 and 4 per group, respectively) or (**E**) GHRH mRNA levels in hypothalamus of 10 days old normally fed or underfed females (n = 10 and 9 per group, respectively). Data are presented as the mean ± SEM. Gene expression determinations are normalized against the histone H3 gene (**C**, **E**). Comparisons were performed by repeated measure two-way ANOVA analysis (**A**) or Mann Whitney analysis (**B**–**E**).
(TIF)

S2 Fig GHRH receptor and Pit-1 expression levels in adult male mice. (**A**) GHRH R mRNA levels in pituitary of adult (3 month-old) male mice previously normally fed or underfed (n = 6 and 7 per group, respectively). (**B**) Pit-1 mRNA levels in pituirary of same animals (n = 7 per group). Data are presented as the mean ± SEM. Gene expression determinations are normalized against the histone H3 gene. Comparisons were performed with Mann Whitney test.
(TIF)

S3 Fig Nutritional Restriction is not associated with a decreased density of lactotroph cells population in pituitary of 20 day-old male pups. Immunohistochemistry against prolactin (PRL) (in red) and counterstained with DAPI (in blue) of pituitary harvested from 20 day-old normally fed or underfed male pups in serial series of slices used for the GH mmunohistochemistry (see Fig 1D) indicates that the density of lactotroph cells that produce PRL are not decreased with underfeeding and even tend to increase (not significant). Representative micrograph are presented for normally fed (left panels) and underfed (midle panel), and the quantification is presented in the right panel. Data are presented as the mean ± SEM. Comparisons were performed with a Mann Whitney analysis.
(TIF)
