# Correction: IGF-1 Induces GHRH Neuronal Axon Elongation during Early Postnatal Life in Mice

**DOI:** 10.1371/journal.pone.0172915

**Published:** 2017-02-21

**Authors:** Lyvianne Decourtye, Erik Mire, Maud Clemessy, Victor Heurtier, Tatiana Ledent, Iain C. Robinson, Patrice Mollard, Jacques Epelbaum, Michael J. Meaney, Sonia Garel, Yves Le Bouc, Laurent Kappeler

The Data Availability statement for this paper is incorrect. The correct statement is: Data are available from the figshare repository (https://doi.org/10.6084/m9.figshare.4629754.v1).
