# Supplementary figures and images for: IGF-1 Induces GHRH Neuronal Axon Elongation during Early Postnatal Life in Mice
Source: PLoS One. 2017 Jan 11;12(1):e0170083. doi: 10.1371/journal.pone.0170083 (PMC5226784; doi:10.1371/journal.pone.0170083)

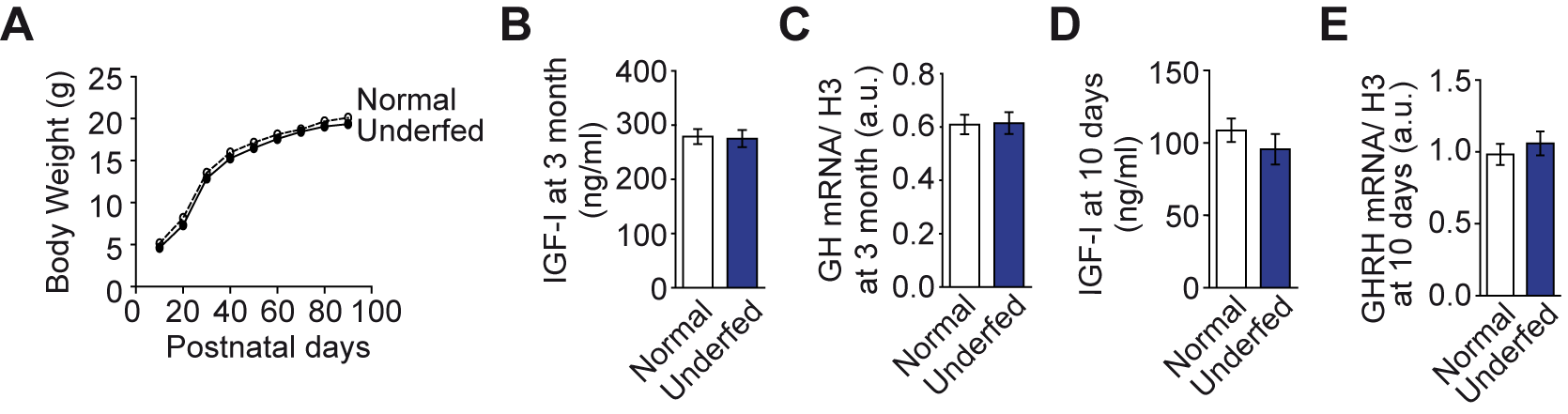

Supplement: S1 Fig — (A) Body weight gain during postnatal development is similar between normally fed (n = 17–22) and underfed females (n = 23–25). Coherently, (B) plasma IGF-1 circulating levels (n = 10 per group) and (C) GH mRNA levels in pituitary (n = 9 per group) at 3month of age are similare between females that have been previously normally fed or underfed during lactation. Moreover, Nutritional restriction is not associated with a decrease of (D) plasma IGF-1 circulating levels at 10 days of age (n = 6 and 4 per group, respectively) or (E) GHRH mRNA levels in hypothalamus of 10 days old normally fed or underfed females (n = 10 and 9 per group, respectively). Data are presented as the mean ± SEM. Gene expression determinations are normalized against the histone H3 gene (C, E). Comparisons were performed by repeated measure two-way ANOVA analysis (A) or Mann Whitney analysis (B–E). (TIF) [file pone.0170083.s001.tif]

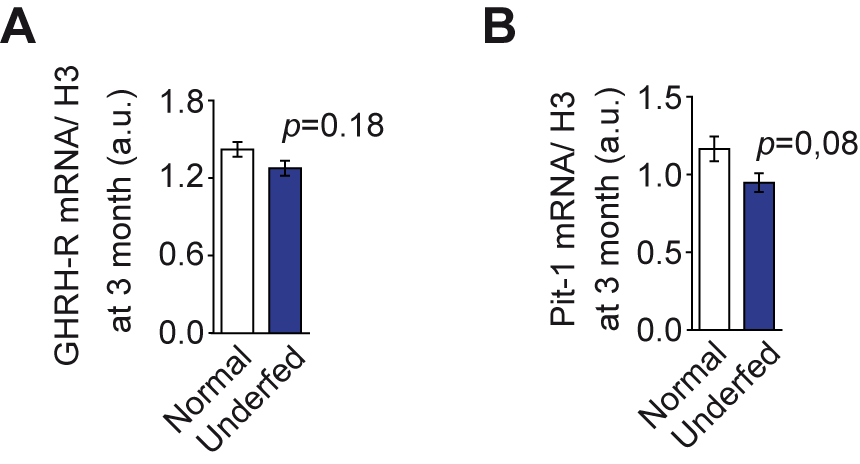

Supplement: S2 Fig — (A) GHRH R mRNA levels in pituitary of adult (3 month-old) male mice previously normally fed or underfed (n = 6 and 7 per group, respectively). (B) Pit-1 mRNA levels in pituirary of same animals (n = 7 per group). Data are presented as the mean ± SEM. Gene expression determinations are normalized against the histone H3 gene. Comparisons were performed with Mann Whitney test. (TIF) [file pone.0170083.s002.tif]

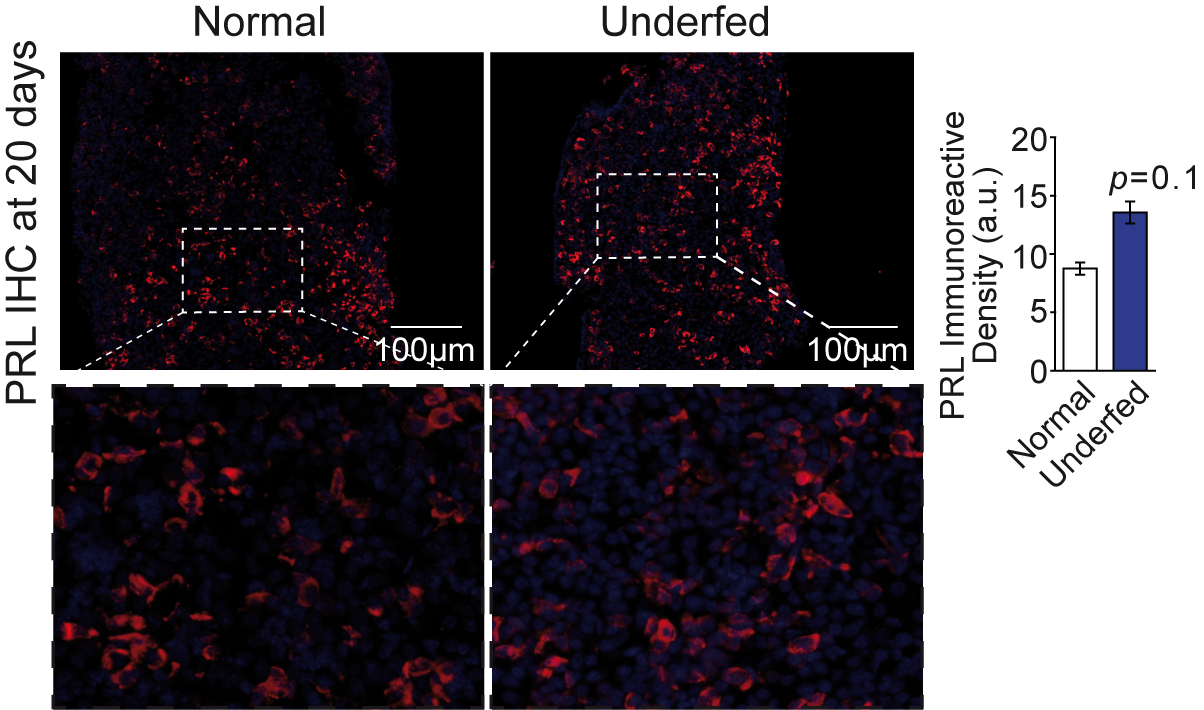

Supplement: S3 Fig — Immunohistochemistry against prolactin (PRL) (in red) and counterstained with DAPI (in blue) of pituitary harvested from 20 day-old normally fed or underfed male pups in serial series of slices used for the GH mmunohistochemistry (see Fig 1D) indicates that the density of lactotroph cells that produce PRL are not decreased with underfeeding and even tend to increase (not significant). Representative micrograph are presented for normally fed (left panels) and underfed (midle panel), and the quantification is presented in the right panel. Data are presented as the mean ± SEM. Comparisons were performed with a Mann Whitney analysis. (TIF) [file pone.0170083.s003.tif]
